# Spatiotemporal regulation of the BarA/UvrY two-component signaling system

**DOI:** 10.1016/j.jbc.2023.104835

**Published:** 2023-05-17

**Authors:** Fernanda Urias Contreras, Martha I. Camacho, Archana Pannuri, Tony Romeo, Adrian F. Alvarez, Dimitris Georgellis

**Affiliations:** 1 Departamento de Genética Molecular, Instituto de Fisiología Celular, Universidad Nacional Autónoma de México, México D.F., México; 2 Department of Microbiology and Cell Science, University of Florida, Gainesville, Florida, USA

**Keywords:** Escherichia coli, two component system, BarA, HflKC complex, spatiotemporal regulation

## Abstract

The BarA/UvrY two-component signal transduction system mediates adaptive responses of *Escherichia coli* to changes in growth stage. At late exponential growth phase, the BarA sensor kinase autophosphorylates and transphosphorylates UvrY, which activates transcription of the CsrB and CsrC noncoding RNAs. CsrB and CsrC, in turn, sequester and antagonize the RNA binding protein CsrA, which posttranscriptionally regulates translation and/or stability of its target mRNAs. Here, we provide evidence that during stationary phase of growth, the HflKC complex recruits BarA to the poles of the cells and silences its kinase activity. Moreover, we show that during the exponential phase of growth, CsrA inhibits *hflK* and *hflC* expression, thereby enabling BarA activation upon encountering its stimulus. Thus, in addition to temporal control of BarA activity, spatial regulation is demonstrated.

The BarA (bacterial adaptive response) protein, a membrane-bound tripartite sensor kinase (1, 2), and the UvrY protein, a typical response regulator of the FixJ family (3), constitute a two-component signaling system in *Escherichia coli* that mediates adaptative responses by modulating the Csr global regulatory system (4). BarA senses and responds to the presence of the protonated form of short-chain carboxylic acids such as formate and acetate (5, 6, 7), at the late exponential phase of growth, leading to its autophosphorylation and transphosphorylation of the cognate response regulator UvrY (3, 8, 9). UvrY-P, in turn, activates transcription of the CsrB and CsrC noncoding regulatory RNAs (4, 10, 11, 12) that possess repeated sequence elements enabling them to interact with multiple copies of the RNA-binding protein CsrA and thereby antagonize its regulatory functions, by preventing the interaction of CsrA with its mRNA targets (13, 14).

CsrA, an RNA binding protein, posttranscriptionally coordinates gene expression by interacting with its target RNAs at sites characterized by a conserved GGA sequence, typically located within 5′ untranslated mRNA leaders, and positively or negatively regulates RNA stability, translation, or transcription elongation (15, 16, 17, 18, 19, 20). In this way, CsrA activates exponential phase processes while represses several stationary phase functions (21, 22). CsrA is widely distributed among eubacteria (11, 23, 24) and regulates expression of genes for virulence factors (25, 26, 27), quorum sensing (28, 29), motility (30, 31), carbon metabolism (32, 33), biofilm formation (34, 35, 36), cyclic di-GMP synthesis (37), iron homeostasis (38) and peptide uptake (17).

In this study, we provide evidence that during stationary phase of growth, the HflKC complex recruits BarA to the poles of the cells, leading to the inhibition of BarA-dependent gene expression. Moreover, we show that CsrA negatively affects HflK and HflC expression during the exponential phase of growth. This allows BarA activation in the presence of its stimulus, at the transition from exponential to stationary phase of growth. Our findings are incorporated into a complex model for the BarA/UvrY-CsrA/B/C circuitry to include, in addition to temporal control, a spatial regulation of the BarA activity.

## Results

### BarA interacts with HflK and HflC

It has been previously reported that activation of the BarA sensor kinase, which normally takes place at the transition from the exponential to stationary phase of growth (4), does not occur in a CsrA mutant strain (39). The effect of CsrA on BarA was suggested to be indirect, and therefore, it was proposed that in addition to acetate, which acts as its physiological stimulus for BarA (5, 6), other factors are involved in the control of the activity of BarA (39). To identify such factors, a pull-down experiment using BarA as the bait was performed. Briefly, a *barA* mutant strain (IFC5035) harboring plasmid pMX559, which carries a His_6_-tagged BarA version under the arabinose promoter, was grown to an absorbance at 600 nm (*A*_600_) of ∼1.0, and 0.13 mM of arabinose was added to induce BarA expression. After an hour of induction, the protein crosslinker formaldehyde was added for 10 min. As a control, the same procedure was pursued for the *barA* mutant strain but without the His_6_-BarA-expressing plasmid. Subsequently, cells were harvested, lysed by French Press, BarA was purified under denaturing conditions by nickel affinity chromatography, the crosslinking was reversed, and proteins that copurified with BarA were identified by LC-MS/MS analyses (Table S1). After eliminating the proteins that appeared in the control experiments and the cytosolic proteins, HflK emerged as the highest hit (Table S1). HflK, which is encoded on the *hfq-hflXKC* operon, is an inner membrane protein that forms part of the HflKC complex. This complex interacts with and regulates the ATP-dependent protease FtsH (40, 41).

**Figure 1:**
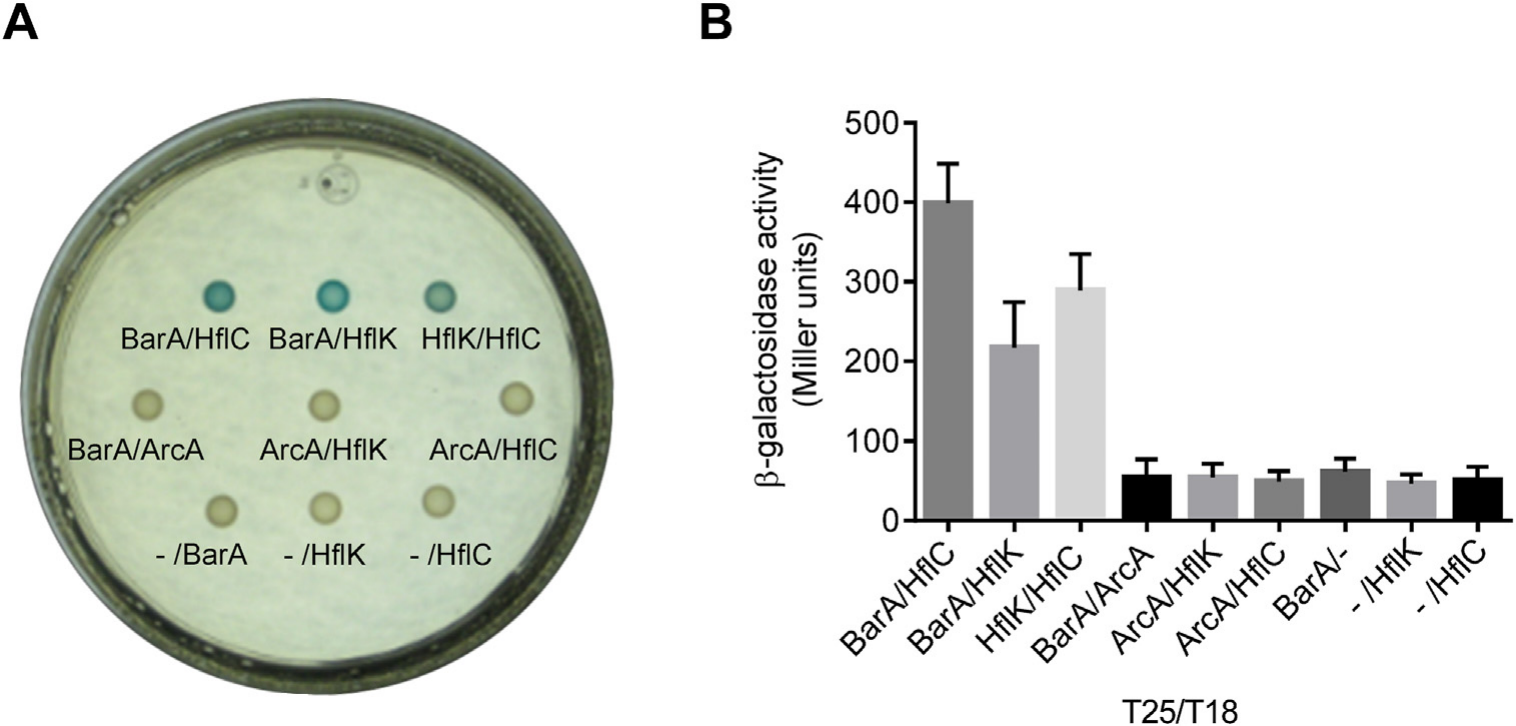
**BarA interacts with HflK and HflC.** Strain BTH101 (*cya*^−^) was cotransformed with recombinant plasmids expressing BarA, HflK, HflC, or ArcA hybrid proteins fused to either the T25 or the T18 domain of adenylate cyclase. *A*, bacterial cultures were spotted on a X-gal and IPTG containing agar plates, and transcriptional activation of the *lac* operon, indicative of protein interaction, was visualized by the formation of blue colonies. *B*, transformants were grown in LB medium supplemented with 0.5 mM IPTG to an *A*_600_ of 0.6, and β-galactosidase activity was measured. The average from three independent experiments is presented, and standard deviations (error bars) are indicated. LB, lysogeny broth; X-gal, 5-bromo-4-chloro-3-indolyl-β-D-galactoside.

To assure that HflK indeed interacts with BarA *in vivo*, the bacterial adenylate cyclase-based two hybrid system (42) was employed. For this purpose, the T25 or the T18 subunit, corresponding to amino acids 1 to 224 or 225 to 399 of the adenylate cyclase (CyaA), respectively, was fused to the N terminus of BarA, HflK, HflC, and ArcA (see Experimental procedures) to generate plasmids pT25BarA, pT25HflK, pT18HflK, pT25ArcA, pT18ArcA, and pT18HflC (Table S2). Interaction between two hybrid proteins leads to reconstitution of the catalytic domain of the adenylate cyclase resulting in restoration of cAMP production in *E. coli cya* mutants and thereby activation of, among others, the lactose operon. The activation of this operon can be detected on selective agar plates or using β-galactosidase assays. It is relevant to mention that bacterial adenylate cyclase–based two hybrid system has been shown to detect interactions between not only cytoplasmic proteins but also between membrane-associated proteins (43). *E. coli* BTH101, a *cya* mutant, was cotransformed with pairs of the recombinant plasmids of interest, and bacteria were plated on lysogeny broth (LB) + 5-bromo-4-chloro-3-indolyl-β-D-galactoside (X-gal) plates (Fig. 1*A*). It was observed that when BTH101 was cotransformed with the pT25BarA/pT18, pT25/pT18HflK, pT25/pT18HflC, pT25BarA/pT18ArcA, pT25ArcA/pT18HflK, or pT25ArcA/pT18HflC vectors yielded colorless colonies. On the other hand, when BTH101 was cotransformed with pT25HflK/pT18HflC, which serves as positive control, or with pT25BarA/pT18HflK and pT25BarA/pT18HflC yielded blue colonies. This indicates heterodimerization of the chimeric proteins, resulting in efficient functional complementation of the adenylate cyclase fragments.

The efficiency of functional complementation between T25 and T18 domains was then quantified by measuring β-galactosidase activity (Fig. 1*B*) of liquid cultures. It was observed that the β-galactosidase activity in cells harboring pT25HflK/pT18HflC, pT25BarA/pT18HflK, and pT25BarA/pT18HflC was significantly higher than in cells harboring pT25BarA/pT18, pT25/pT18HflK, pT25/pT18HflC, pT25BarA/pT18ArcA, pT25ArcA/pT18HflK, or pT25ArcA/pT18HflC (Fig. 1*B*). It can therefore be concluded that BarA does interact with the HflKC complex *in vivo*.

### BarA colocalizes with the HflKC complex *in vivo*

To provide further evidence for the interaction of BarA with the HflKC complex, a strain (IFC5043 pMX560) harboring the BarA-Yfp and HflK-mCherry hybrid proteins was constructed, and the localization/colocalization of the proteins was monitored. It has to be mentioned that a plasmid-born arabinose-inducible BarA-Yfp (pMX560) was used, because the chromosomal BarA-Yfp hybrid was not detectable, most likely due to its low expression. On the other hand, the HflK-mCherry fusion was introduced into the wildtype *hflK* chromosomal locus, and therefore, its expression relies on the native promoter.

**Figure 2:**
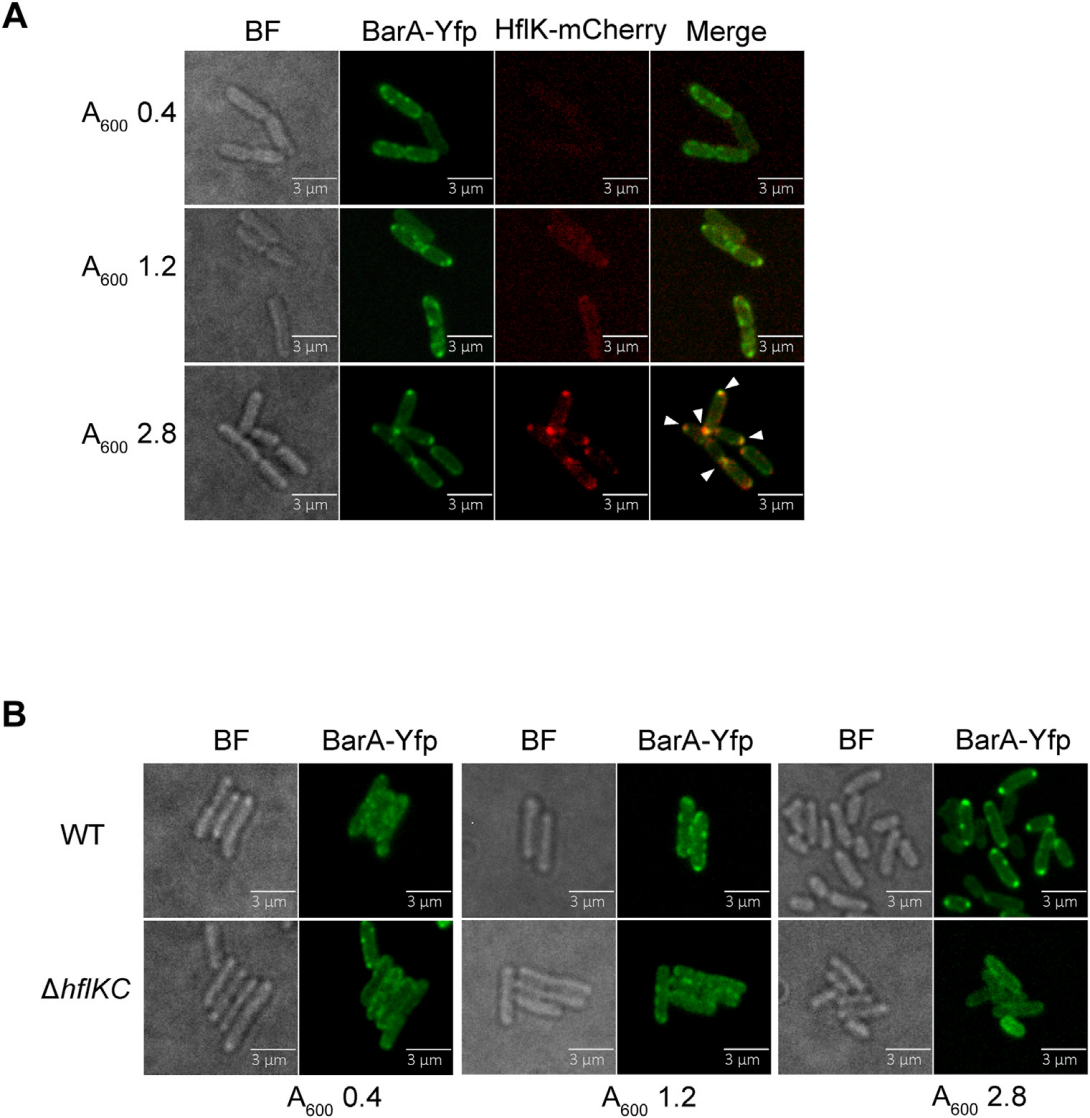
**HflK is required for the recruitment of BarA to the cell poles at the stationary phase of growth.***A*, representative fluorescence images of *E. coli* live cells expressing BarA-mEyfp (colored in *green*) and HflK-mCherry (colored in *red*) translational fusions (strain IFC5043 harboring pMX560 plasmid), harvested during the exponential growth phase (*A*_600_ of 0.4), the early stationary phase (*A*_600_ of 1.2), or during the stationary phase (*A*_600_ of 2.8). *Left panels* show the bright field (BF) imaging of cells; *right panels* show the merged fluorescence signals, which appear *yellow* where the two fluorescence signals overlapped. *Triangles* indicate colocalized foci. *B*, effect of the in-frame deletion of *hflKC* in the BarA-mEyfp localization. Representative bright field (BF) and fluorescence images of CF7789 (WT) and IFC5047 (Δ*hflKC*::Kan^r^) cells, expressing BarA-mEyfp (colored in *green*) from plasmid pMX560, harvested at exponential growth phase (*A*_600_ of 0.4, *left panels*), early stationary phase (*A*_600_ of 1.2, *middle panels*) or at stationary phase (*A*_600_ of 2.8, *right panels*).

Fluorescence microscopy of live cells revealed that the intensity of fluorescence of HflK-mCherry was very low in exponential growing cells and significantly higher in cells from the stationary phase of growth (Fig. 2*A*), suggesting that the expression of *hflK* is growth stage dependent. Also, HflK-mCherry was found to localize in discrete foci on cell poles or on the septum region of the cells, as previously reported for its protein complex partner HflC (44, 45). On the other hand, BarA-Yfp was found to form discrete foci that were scattered on the cytosolic membrane of exponential growing cells (*A*_600_ 0.4) but were progressively recruited to the cell poles as the cell density increased (*A*_600_ 1.2) (Figs. 2*A*, and S1). Finally, during the stationary phase of growth (*A*_600_ 2.8), BarA-Yfp was found to almost exclusively localize on the cell poles, colocalizing with HflK (Figs. 2*A* and S1). As expected, a similar topological distribution was observed when the colocalization of HflC-mCherry and BarA-Yfp was analyzed (Fig. S2). Next, we asked whether the HflKC complex is required for the recruitment of BarA to the cell poles at the stationary phase of growth. To this end, the *in vivo* localization of the BarA-Yfp hybrid protein in an *hflK hflC* double mutant (IFC5047) and its isogenic wildtype strain (CF7789) was compared. It was noticed that the BarA-Yfp hybrid protein, in contrast to the polar localization in the wildtype strain, was dispersed throughout the membrane in the *hflK hflC* mutant strain (Fig. 2*B*), indicating that the HflKC complex is required for proper BarA localization. It, thus, appears that the HflKC complex, when present, interacts with BarA and restrains its localization to the poles of the cells.

### CsrA binds to the hflK mRNA and inhibits its translation

**Figure 3:**
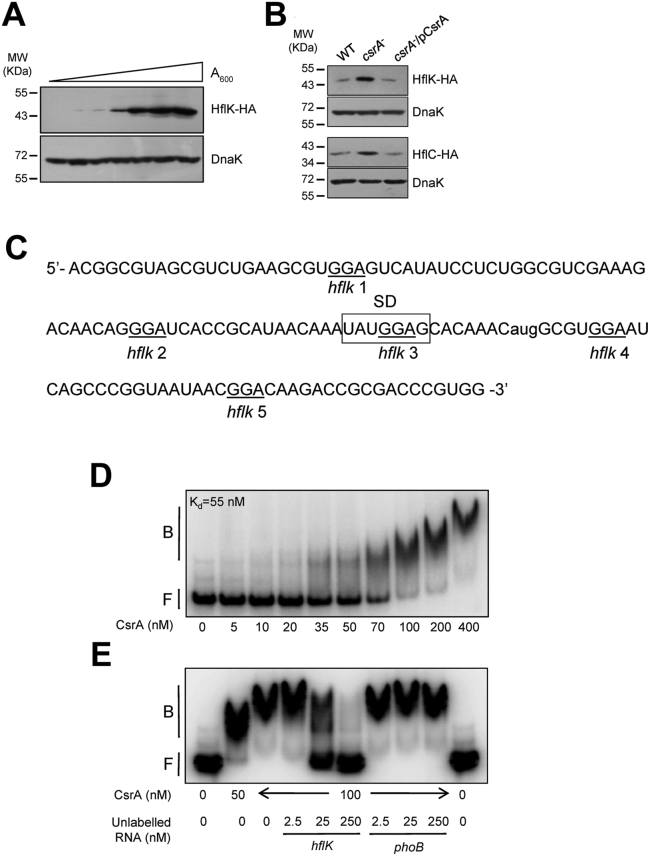
**CsrA modulates the expression of the HflKC complex.***A*, levels of HflK-HA protein (46.8 KDa) as determined by Western blot analysis. A culture of strain IFC5021 (*hflK*::*ha*) was grown in LB medium, cells were harvested by centrifugation throughout the growth curve (*A*_600_ of 0.3–3.0), and HflK-HA content was determined by Western blot using monoclonal antibodies against the HA epitope. DnaK (69.1 kDa), detected using DnaK polyclonal antibodies, was used as a loading control. *B*, (*upper panels*) levels of HflK-HA protein (46.8 KDa) in IFC5021 (*hflK*::*ha*) and IFC5054 (Δ*csrA hflK*::*ha*) strains and in IFC5054 (Δ*csrA hflK*::*ha*) complemented with the *csrA*-expressing plasmid pMX544 (indicated as pCsrA), as determined by Western blot analyses using monoclonal antibodies against HA; (*lower panels*). Levels of HflC-HA protein (38.9 KDa) in the IFC5019 (*hflC*::*ha*) and IFC5055 (Δ*csrA hflC*::*ha*) strains and in IFC5055 (Δ*csrA hflC*::*ha*) complemented with the *csrA*-expressing plasmid pMX544 (indicated as pCsrA), as determined by Western blot analyses using monoclonal antibodies against HA. DnaK (69.1 kDa), detected using DnaK polyclonal antibodies, was used as a loading control. Cultures of the indicated strains were grown in LB medium, and at an *A*_600_ of 0.5, cells were harvested for Western blot analysis. Experiments were repeated three times in their entirety with essentially identical results. *C*, nucleotide sequence of the *hflK* RNA (134 nt) used in EMSA’s comprised of RNA extending from −86 to +48 nt with respect to the start codon. The GGA sequences are underlined, and the start codon is given in lowercase letters. The Shine–Dalgarno (SD) sequence is *boxed*. *D*, gel shift analysis of CsrA binding to mRNA leader of *hflK*. 5′ end-labeled RNA (0.5 nM) was incubated with the concentration of CsrA indicated at the *bottom* of each lane. Kd value of the CsrA interaction with the *hflK* transcript is shown. *E*, RNA competition assay. Labeled *hflK* leader RNA (0.5 nM) was incubated with CsrA ± specific (*hflK*) or nonspecific (*phoB*) competitor RNA. Positions of free (F) and bound (B) RNA are indicated. LB, lysogeny broth.

To confirm that *hflK* expression is growth stage dependent, as suggested above (Fig. 2*A*), the amount of HflK was monitored throughout the growth curve of IFC5021, carrying a chromosomal HflK-HA protein fusion (Fig. 3*A*). Indeed, the amount of HflK was almost undetectable during exponential growth and increased significantly at the early stages of the stationary phase of growth (Fig. 3*A*). Because the expression of the *hfq-hfl*XKC operon is not known to be transcriptionally activated during stationary phase of growth, the nucleotide sequence in the vicinity of the *hflK* start codon was inspected for possible regulatory motifs. Various possible CsrA binding sites (*hflk* 1–5), characterized by the GGA sequence (46, 47), located at −65, −32, −11, +7, and +27 relative to the start codon of HflK (Fig. 3*C*) were found. Comparison of the GGA sequences and flanking regions with the reported consensus (Fig. S3) revealed good match of *hflk* 2–4 to consensus sequences. However, folding prediction of the mRNA region spanning nucleotides −86 to +48 relative to the start codon of the *hflK* mRNA (Fig. S4) places *hflk* 2 in a base-paired region, which should prevent CsrA interaction. To test whether CsrA is indeed involved in the growth-dependent regulation of *hflK* expression, the amount of HflK in a wildtype (IFC5021) and an isogenic *csrA*::*kan* mutant (IFC5054) strains, harboring a chromosomal HflK-HA fusion, was compared by Western blotting, using specific HA antibodies. It was found that the amount of HflK was significantly higher in the *csrA* mutant strain than in the wildtype strain (Fig. 3*B*). The level of HflK protein was re-established at wildtype levels when the *csrA* mutant strain was complemented with a *csrA*-expressing plasmid (pMX544) (Fig. 3*B*), indicating that CsrA inhibits, directly or indirectly, *hflK* expression. Similar results were obtained for an HflC-HA hybrid (Fig. 3*B*).

Finally, the question whether CsrA interacts directly with the mRNA region spanning nucleotide −86 to +48 (134 nt) relative to the start codon of the *hflK* mRNA (Fig. 3*D*) was addressed, by electrophoretic mobility shift assays. It was observed that the intensity of a band with slower migration began to increase at ∼35 nM CsrA, which is indicative of CsrA-hflK RNA complex formation. This complex became more prominent as the CsrA concentration increased further, at the expense of free *hflK*. A linear regression analysis of the data for this interaction yielded an apparent dissociation constant (Kd) of 55 nM (Fig. S5). Also, it was noted that the shifted band migrated progressively slower with increasing amounts of CsrA, suggesting that multiple CsrA proteins may bind to the hflK RNA. Finally, unlabeled *hflK* RNA was able to compete for the formation of CsrA complexes with the labeled *hflK* RNA (Fig. 3*E*), while unlabeled *phoB* RNA, which does not bind to CsrA (48), did not compete with the *hflK* RNA. It can therefore be concluded that CsrA directly binds to *hflK* mRNA and inhibits *hflK* expression (Fig. 3*B*). Because the GGA site at −11 overlaps the Shine–Dalgarno sequence of this mRNA (Figs. 3*C* and S4), CsrA binding is predicted to inhibit ribosome loading and *hflK* translation, the predominant inhibitory mode of CsrA (22). However, we did not further investigate the CsrA-dependent inhibitory mechanism.

### The HflKC complex silences the BarA kinase activity

**Figure 4:**
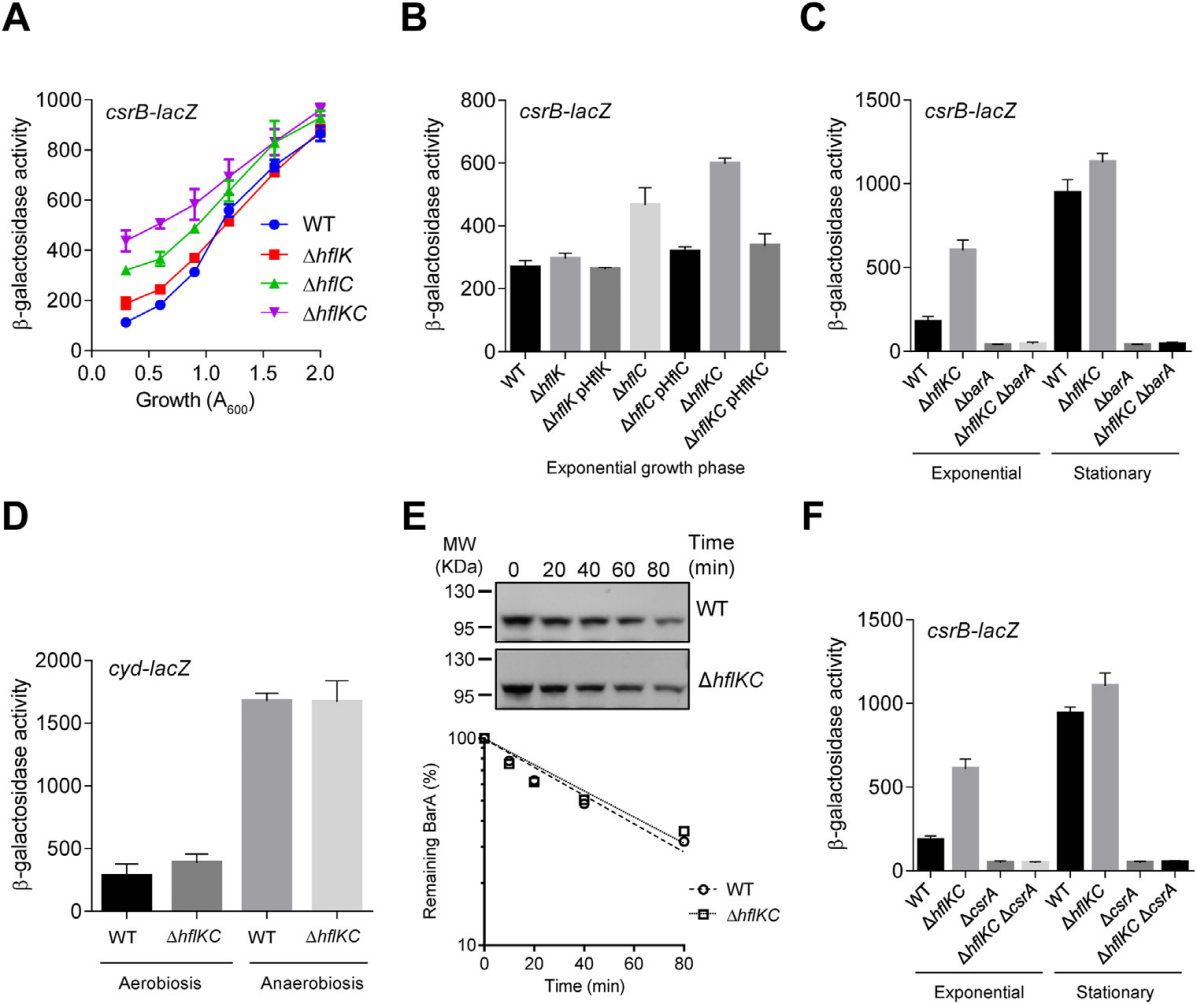
**The HflKC complex negatively affects the BarA kinase activity.***A*, cells of the *csrB-lacZ* transcriptional fusion-carrying strains KSB837 (WT) (*blue*, *filled circles*), IFC5051 (Δ*hflK*) (*red*, *filled squares*), IFC5052 (Δ*hflC*) (*green*, *filled up-pointing triangle*), and IFC5053 (Δ*hflKC*) (*violet*, *filled down-pointing triangle*) were harvested at various times throughout growth and assayed for β-galactosidase activity. The β-galactosidase activity is presented as a function of growth density (*A*_600_). Data represent the averages from three independent experiments, and standard deviations (error bars) are indicated. *B*, strains KSB837 (WT), IFC5051 (Δ*hflK*), IFC5052 (Δ*hflC*), IFC5053 (Δ*hflKC*), IFC5051 (Δ*hflK*) carrying plasmid pT25HflK (pHflK), IFC5052 (Δ*hflC*) carrying plasmid pT25HflC (pHflC), and IFC5053 (Δ*hflKC*) carrying plasmid pMX561 (indicated as pHflKC and expressing *hflK* and *hflC*) were grown in LB medium to an *A*_600_ of ∼0.6 (exponential phase) and β-galactosidase activity was measured. The average from three independent experiments is presented, and standard deviations (error bars) are indicated. *C*, strains KSB837 (WT), IFC5053 (Δ*hflKC*), IFC5035 (Δ*barA*), and IFC5058 (Δ*hflKC* Δ*barA*) were grown in LB medium to an *A*_600_ of 0.6 (exponential phase) or 2.5 (stationary phase), and β-galactosidase activity was measured. The average from three independent experiments is presented, and standard deviations (error bars) are indicated. *D*, strains ECL5003 (*cyd-lacZ*) and its isogenic IFC5057 (Δ*hflKC*::Kan^r^*cyd-lacZ*), carry the ArcA-P−activatable *cydA-lacZ* reporter, were grown aerobically (nonstimulatory conditions) or anaerobically (stimulatory conditions) in LB medium to an *A*_600_ of 0.6, and β-galactosidase activity was measured. The average from three independent experiments is presented, and standard deviations (error bars) are indicated. *E*, determination of stability of the BarA protein, after inhibition of protein synthesis with tetracycline, in a wildtype strain (CF7789) and in a *hflKC* mutant strain (IFC5047). (*Top*) Western blot analysis using BarA polyclonal antibodies. (*Bottom*) Semilogarithmic plot of BarA protein decay. *F*, β-galactosidase activities of KSB837 (WT), IFC5053 (Δ*hflKC*), IFC5010 (Δ*csrA*), and IFC5056 (Δ*hflKC* Δ*csrA*) cells grown in LB medium to an *A*_600_ of 0.6 (exponential phase) or 2.5 (stationary phase). The average and standard deviations from three independent experiments are presented. LB, lysogeny broth.

We then asked whether the activity of BarA is affected in an *hflK*, *hflC*, or a double *hflKC* mutant. To this end, the expression of the *csrB-lacZ* reporter, which depends directly on the activity of the BarA/UvrY TCS, was monitored in a wildtype strain (KSB837) and the isogenic Δ*hflK* (IFC5051), Δ*hflC* (IFC5052), and Δ*hflKC* (IFC5053) mutant strains (see Experimental procedures). It was found that, during exponential growth, reporter expression was slightly higher in the Δ*hflK* mutant strain, and approximately 3- and 4-fold higher in the Δ*hflC* and Δ*hflKC* mutant strains, respectively (Fig. 4*A*). Reporter expression was restored to almost wildtype levels when these mutant strains were complemented with the corresponding HflK (pT25HflK), HflC (pT25HflC), and HflKC (pMX561) expressing plasmids (Fig. 4*B*). It can therefore be concluded that the HflKC complex negatively affects the BarA/UvrY signaling system. The effect of HflKC was most likely exerted through BarA, because no activation of *csrB-lacZ* expression was obtained in an *hflK hflC barA* triple mutant (IFC5058) (Fig. 4*C*). To examine whether the HflKC complex affects other two-component sensor kinases, the activity of ArcB, which is activated during anoxic growth conditions (49, 50), in an Δ*hflKC* mutant (IFC5057) and the corresponding isogenic wildtype strain (ECL5003) was tested, by monitoring the expression of the activatable *cyd-lacZ* reporter (49). No difference in reporter expression was noted between the wildtype and the *hflKC* mutant strains under aerobic or anoxic growth conditions (Fig. 4*D*), indicating that the HflKC complex specifically affects the BarA sensor kinase.

As mentioned earlier, FtsH, an ATP-dependent metalloprotease, whose activity is modulated by the HflKC complex, degrades a number of soluble and inner membrane proteins (51). However, the fact that the HflKC complex antagonizes the FtsH proteolytic activity (41) in combination with our result demonstrating a BarA gain-of-function phenotype in the *hflKC* mutant excludes the possibility that the observed effect is due to the degradation of BarA. At any rate, this possibility was explored by comparing the half-life of BarA in an Δ*hflKC* mutant (IFC5047) and its wildtype isogenic strain (CF7789). No significant difference in the stability of BarA in these two strains was found (Fig. 4*E*). This is in agreement with a previous finding that no difference in the amount of BarA was found between a wildtype strain and a *csrA* mutant where expression of *hflK* and *hflC* are not repressed (39). Therefore, it can be concluded that the HflKC complex exerts a direct effect on the BarA kinase activity.

The facts that the HflKC complex binds to BarA (Figs. 1 and 2) and inhibits its kinase activity, as judged by *csrB-lacZ* expression (Fig. 4*A*) and that CsrA inhibits *hflK* expression (Fig. 3), prompted us to ask whether deletion of *hflK* and *hflC* in a *csrA* mutant could lead to activation of BarA. No activation of *csrB-lacZ* was detected in a *csrA hflK hflC* triple mutant (IFC5056) (Fig. 4*F*), suggesting that additional factors are involved in the regulation of the BarA kinase activity.

### HflKC acts in the stationary phase of growth

**Figure 5:**
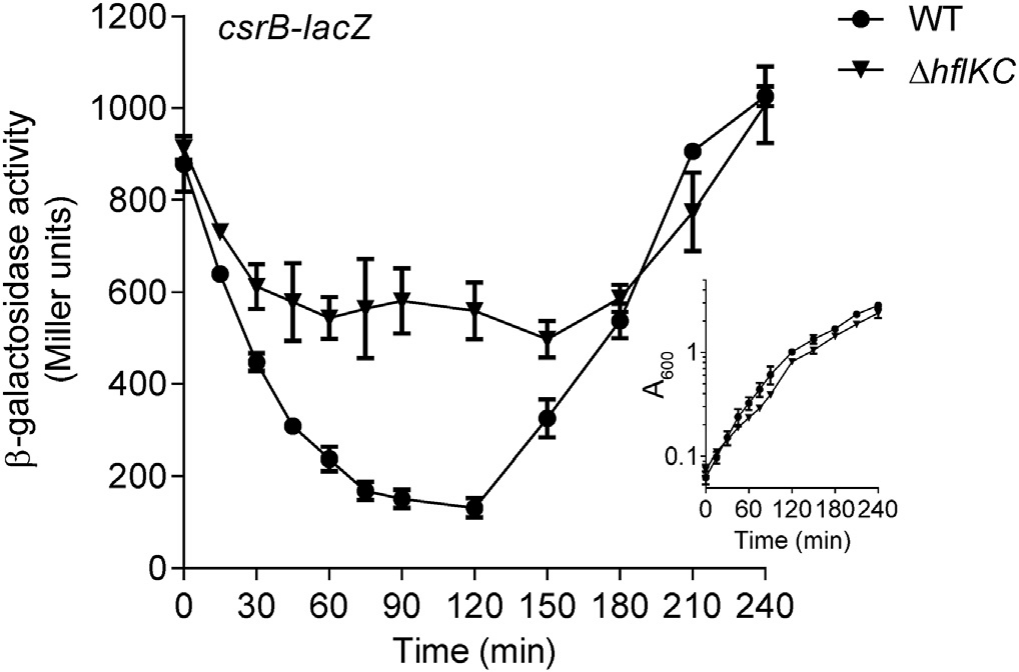
**The BarA inhibition by the HflKC complex takes place at the stationary phase of growth.** Cultures of strain KSB837 (WT) and its isogenic IFC5053 (Δ*hflKC*), both carrying the UvrY-P activatable *csrB-lacZ* reporter, were grown to the stationary phase of growth, shifted to exponential growth by diluted with fresh LB medium to an *A*_600_ of 0.05, and β-galactosidase activity and cell growth (insert) were followed for 240 min. LB, lysogeny broth.

The result demonstrating that the HflKC complex inhibits the activity of BarA during exponential growth (Fig. 4*A*) appears to be inconsistent with the time of activation of HflK and HflC expression, which takes place during stationary phase of growth (Figs. 2*A*, S1, and 3, *A* and *B*). A possible explanation could be that BarA inhibition by the HflKC complex takes place during the stationary phase of growth and is inherited by the exponentially growing cells when cell growth is resumed. Subsequently, as the cells grow, the existing HflKC complex becomes undetectable, *via* its proteolysis or gradual dilution, permitting the stimulus-dependent activation of BarA at late exponential growth phase. To test this hypothesis, the *hflKC* mutant and its isogenic wildtype strain were grown to the stationary phase of growth, the cultures were shifted to exponential growth by diluting them to an *A*_600_ of 0.05, and the *csrB-lacZ* expression was followed (Fig. 5). It was found that the β-galactosidase activity in the wildtype decreased with time until the culture reached an *A*_600_ of ∼1.0, indicating that BarA remains silent during this period of time (Fig. 5*A*). On the other hand, the β-galactosidase activity in the *hflKC* mutant decreased significantly slower (Fig. 5), indicating that BarA retains some of its kinase activity. It can therefore be concluded that the HflKC complex binds to the BarA sensor kinase and inhibits its kinase activity during the stationary phase of growth. It should be noted that the *hflKC* mutant grows slightly slower than the wildtype strain (Figs. 5 and S6*A*), raising the possibility that the *hflKC* mutant could have a fitness defect. To test this, a competition between the wildtype strain (KSB837, Amp^r^) and the isogenic Δ*hflKC* mutant strain (IFC5047, Kan^r^) was pursued. Equal number of KSB837 and IFC5047 cells, corresponding to an *A*_600_ of 0.01 each, were inoculated into LB medium, and the number of wildtype cells (ampicillin resistant-CFUs/ml) and mutant cells (kanamycin resistant-CFUs/ml) was monitored throughout the growth curve. Under this condition, the wildtype strain outcompeted the *hflKC* mutant strain, becoming the predominant species in the culture as early as 100 min postincubation and reaching almost 75% of the total bacteria after 8 h (Fig. S6*B*). This result highlights the significance of the BarA/UvrY-Csr-HflKC regulatory loop in maintaining bacterial fitness.

## Discussion

We previously reported that the global translational regulator CsrA is required for BarA to activate as a histidine kinase even in the presence of the physiological BarA stimulus (39) and speculated that other factors could participate in the regulation of the activity of the BarA/UvrY TCS. Here, we provide evidence that, during stationary phase of growth, the HflK and HflC proteins, whose expression was found to be regulated by CsrA, bind and recruit BarA to the cell poles, leading to its inactivation. The HflK and HflC proteins have been shown to form a heteromultimer that interacts with and negatively modulates the FtsH protease, which degrades a group of short-lived or misfolded membrane proteins (41, 52, 53). However, the inhibitory effect of the HflKC complex on BarA appears to be independent of the FtsH protease activity, because the BarA stability was not affected in a *hflK hflC* mutant strain. Nonetheless, it cannot be ruled out that FtsH influences the stability of other downstream regulatory components. It is of interest to mention that both the HflK and HflC proteins contain a functional SPFH domain (for Stomatin/Prohibitin/Flotillin/HflK/C), which is found in proteins that are invariant components of lipid rafts or membrane microdomains in eukaryotic and prokaryotic cells (54, 55). SPFH-containing proteins are thought to be involved in protein recruitment to the lipid rafts (54, 56) and have been found to be spatially and functionally associated with signaling pathways in bacteria (55). Indeed, HflK and HflC were recently identified in lipid raft-like membrane microdomains in *E. coli* (45). It is therefore tempting to speculate that the HflKC-dependent BarA recruitment to polar membrane microdomains provides the spatial context for modulating the BarA activity. This hypothesis is consistent with the fact that other protein factors, along the HflKC complex, appear to be involved in the regulation of the BarA kinase activity, since BarA is not active in a *csrA hflK hflC* triple mutant strain.

**Figure 6:**
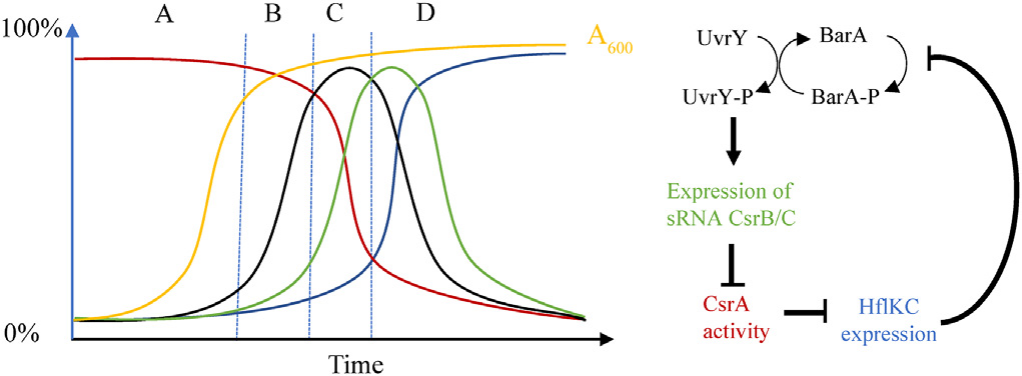
**Physiological model for the BarA/UvrY TCS regulation.** During exponential growth (*A*), BarA activity (*black*) remains low due to the absence of the physiological stimulus and acts as a UvrY-P phosphatase, CsrB and CsrC are not transcribed, and CsrA (*red*) remains free and active, repressing the *hflK* and *hflC* translation (*blue*). At the transition from exponential to stationary phase of growth (*B*), the acetate concentration increases, and the BarA/UvrY TCS becomes active. *C*, the phosphorylated form of UvrY (UvrY-P) activates CsrB expression (*green*), which sequesters and inactivates CsrA (*red*). *D*, CsrA inactivation triggers HflK and HflC expression (*blue*), which localize in the cell poles and recruits BarA, leading to its inactivation. When cell growth resumes (*A*), the existing HflKC complex becomes gradually diluted, permitting the stimulus-dependent activation of BarA at late exponential growth phase.

Nevertheless, the following physiological model (Fig. 6) can be put forward: (A) during exponential growth, BarA remains silent due to the absence of the physiological stimulus (5, 6), and CsrA exerts its regulatory functions, including inhibition of *hflK* and *hflC* expression; (B) the stimulus-dependent activation of BarA occurs at the transition to stationary phase (5, 6), leading to phosphorylation of UvrY, which initiates transcription of *csrB/C* sRNAs (3, 4); (C) when CsrB/C accumulate, they bind to and inhibit the regulatory functions of CsrA (13); (D) the resulting inactivation of CsrA posttranscriptionally derepress *hflK* and *hflC* expression. The accumulated HflK/C binds to BarA, recruiting it to the cell poles and causing its inactivation late in the stationary phase.

The global regulatory role of the Csr system includes the activation of numerous genes and enzymes required for growth, such as glycolytic genes (47). Inhibition of the BarA-UvrY phosphorylation cascade by HflKC and the resulting inhibition of CsrB/C transcription will increase the availability of free CsrA in the late stationary phase of growth and should poise CsrA for rapid posttranscriptional activation of growth-supporting genes when nutrition becomes available and growth resumes. Moreover, such a feedback-loop mechanism could serve to relieve the energy burden during stationary phase of growth, since neither the BarA/UvrY phosphorylation cascade is active nor the CsrB and CsrC sRNAs are transcribed.

In summary, our results provide evidence for a novel cellular function of the HflKC complex and provide further insights into the BarA/UvrY-CsrA regulatory circuitry of *E. coli*. Several two component regulatory systems have been found to rely on auxiliary proteins to modulate the activity of the HK (57). On the other hand, dynamic localization patterns of bacterial sensors have been previously described (58, 59). Yet, this work reveals a regulatory mechanism that involves temporal and spatial sensor kinase dynamics controlled by an auxiliary protein complex in *E. coli*, reminiscent of the *Caulobacter crescentus* CcKA histidine kinase regulatory mechanism (59, 60).

## Experimental procedures

### Bacterial strains, plasmids, and culture conditions

Bacterial strains and plasmids used in this work are listed in Table S2. To construct strains IFC5043 (*hflK*:*mCherry*-Cm^r^) and IFC5044 (*hflC*:*mCherry*-Cm^r^), the *hflK* and *hflC* genes, respectively, were fused *in-frame* with the *mCherry* gene by using the lambda Red recombinase system (61, 62). Briefly, PCR-amplified DNA fragments, using primers pFluor-hflK-Fw and pKD-hflK-Rv or pFluor-hflC-Fw and pKD-hflC-Rv (the sequence of all oligonucleotides used in PCR amplification reactions are shown in Table S3) and plasmid pMXFL2 (44) as the template, were used to transform cells of the pKD46 carrying CF7789 *E. coli* strain, and recombinants were selected by growth on chloramphenicol-agar plates. Similarly, strains IFC5045 (Δ*hflK*::Kan^r^), IFC5046 (Δ*hflC*::Kan^r^), and IFC5047 (Δ*hflKC*::Kan^r^) were generated by lambda red recombinase-facilitated homologous recombination of PCR-amplified products using primers pair hflK70-Fw/hflK70-Rv and plasmid pKD13 (61) as template, or hflC70-Fw/hflC70-Rv and plasmid pKD13 (61) as template, or hflK70-Fw/hflC-pKD-Rv and pKD4 (61) as template, respectively. Then, the FRT-flanked Kan^r^ cassette was removed from strains IFC5045, IFC5046, and IFC5047 using the Flp recombinase encoded by the temperature-sensitive plasmid pCP20 (63), obtaining strains IFC5048 (Δ*hflK*), IFC5059 (Δ*hflC*), and IFC5050 (Δ*hflKC*), respectively. The *csrB-lacZ-*Ap^r^ allele was transferred from KSB837 (CF7789 *csrB-lacZ*) into strains IFC5048 (Δ*hflK*), IFC5049 (Δ*hflC*), and IFC5050 (Δ*hflKC*), by P1*vir* transduction to obtain strains IFC5051 (Δ*hflK csrB-lacZ*), IFC5021 (Δ*hflC csrB-lacZ*), and IFC5053 (Δ*hflKC csrB-lacZ*), respectively. The *barA*::Kan^r^ allele was transferred from strain IFC5035 (6) to IFC5053 (Δ*hflKC csrB-lacZ*) to obtain IFC5058 (Δ*hflKC* Δ*barA*::Kan^r^ *csrB-lacZ*). Strains IFC5054 (*csrA*::Kan^r^ *hflK*:*ha*-Cm^r^), IFC5055 (*csrA*::Kan^r^ *hflC*:*ha*-Cm^r^), and IFC5056 (Δ*hflKC csrA*::Kan^r^ *csrB-lacZ*) were constructed by transfer of the *csrA*::Kan^r^ allele from strain TR1-5CF7789 (4) into strain IFC5021 (*hflK*:*ha*-Cm^r^) (45), IFC5019 (*hflC*:*ha*-Cm^r^) (44), or IFC5053 (Δ*hflKC csrB-lacZ*), respectively, by P1*vir* transduction. Similarly, strain IFC5057 (Δ*hflKC*::Kan^r^ *cyd-lacZ*) was constructed by P1*vir* transduction of the Δ*hflKC*::Kan^r^ allele from strain IFC5047 (Δ*hflKC*::Kan^r^) into strain ECL5003 (*cyd-lacZ*) (64).

To construct plasmid pMX559, expressing *barA* under the control of the L-arabinose-inducible promoter *ara*, a 1.5 Kb DNA fragment containing the *ara* promoter and the ArcB^521–778^ coding sequence, obtained from plasmid pMX020 (65) by NruI and HindIII digestion, was cloned into the same restriction sites of plasmid pACT3 (66), to obtain pACT3-araP-arcB^521–778^. Subsequently, the *barA* open reading frame was isolated as a 2.8 Kb NdeI-HindIII fragment from the plasmid pUC18-barA (6) and inserted between the NdeI and HindIII sites of the above construct, resulting in the plasmid pMX559. To construct plasmid pUC19-barA-mEYFP, the mEYFP coding sequence was amplified by PCR using the primers Yfpcf1SacI and YfPcr1HindIII and plasmid pYFPC-4 (67) as the template. The PCR product was digested with SacI-HindIII and cloned between the SacI-HindIII sites of plasmid pUC19 (68), resulting in the plasmid pUC19-mEYFP. Then, the *barA* coding sequence was PCR amplified using the primer pair barA-NdeI-Fw/barAr1SacI and plasmid pUC18-barA as the template, and the purified PCR product was digested with NdeI and SacI and cloned into the same restriction sites of pUC19-mEYFP to obtain pUC19-barA-mEYFP. To construct plasmid pMX560, which carries a *barA-mEyfp* fusion under the control of the L-arabinose-inducible promoter *ara*, a 3.5 Kb NdeI-HindIII–restricted DNA fragment, obtained from plasmid pUC19-barA-mEYFP and containing the *barA-mEyfp* fusion, was used to replace the *arcB*^551–778^ coding sequence in plasmid pMX020 (65), resulting in the plasmid pMX560. To construct the low copy plasmid pMX561, expressing *hflK* and *hflC* under the control of the native *hfq*-*hflXKC*-operon promoter, a 755 bp DNA fragment was amplified by PCR using primers hfqP-Fw-Hind and hfqP-Rv and the chromosomal DNA of strain CF7789 as a template and cloned as a HindIII-BamHI-restricted DNA fragment into the same restriction sites of plasmid pEXT21 (66). The resulting plasmid was opened by digestion with NdeI and EcoRI and used to clone a DNA fragment, containing the contiguous *hflK* and *hflC* genes, which was PCR amplified by using the primer pair hflK-ORF-Fw/hflC-Rv and the chromosomal DNA of strain CF7789 as a template and subsequently digested with NdeI and EcoRI restriction enzymes, obtaining plasmid pMX561. To construct plasmids pT25BarA and pT18BarA, containing T25 and T18 N-terminal tagged BarA, respectively, the DNA sequence encoding the full-length *barA* was PCR-amplified by using primers DH-ACBarA-Fw and DH-ACBarA-Rv, and the chromosomal DNA of strain CF7789 as a template. The PCR product was digested with BamHI and EcoRI and cloned into the same restriction sites of plasmid pKT25 or pUT18 C to obtain pT25BarA and pT18BarA, respectively. Similarly, DNA fragments containing the *hflK*, *hflC*, or *arcA* coding sequence, amplified by PCR using primers DH-ACHflK-Fw and DH-ACHflK-Rv, DH-ACHflC-Fw and DH-ACHflC-Rv, or DH-ACArcA-Fw and DH-ACArcA-Rv, respectively, were cloned into the BamHI-EcoRI restriction sites of plasmid pKT25 or pUT18C to construct pT25HflK, pT25HflC, pT25ArcA, pT18HflK, pT18HflC, and pT18ArcA.

Bacteria were routinely cultured at 37 °C in LB medium. When required, the LB medium was buffered at pH 7.0 with 100 mM 3-(N-morpholino)propanesulfonic acid (Mops) and/or supplemented with kanamycin (50 μg/ml), ampicillin (100 μg/ml), or chloramphenicol (20 μg/ml). LB agar medium was prepared by addition of 1.5% (w/v) agar. For the qualitative detection of β-galactosidase activity, LB agar was supplemented with X-gal (40 μg/ml).

### *In vivo* protein crosslinking, purification of protein complexes, and mass spectrometry

*In vivo* formaldehyde crosslinking was carried out as previously described (69), with slight modifications. Briefly, cells of strain IFC5035 (Δ*barA*::Kan^r^) harboring plasmid pMX559 or pACT3 (66), as a control, were grown in 500 ml of LB medium, supplemented with kanamycin and chloramphenicol, in a rotary shaker at 37 °C, and at an *A*_600_ of 1.0, the expression of the His_6_-tagged BarA was induced by the addition of 0.13 mM L-arabinose. After 1 h of induction, formaldehyde was added to the cultures at a final concentration of 1% and cells were incubated for further 10 min at 37 °C. The formaldehyde was inactivated by the addition of ice-cold glycine in PBS at a final concentration of 0.125 mM, cells were incubated at 4 °C for 15 min, harvested by centrifugation at 4000*g* for 10 min, and the cell pellet was resuspended in 10 ml of lysis buffer (100 mM NaH_2_PO_4_, 10 mM Tris/Cl, pH 8.0, 8 M urea). The protein complexes were purified under denaturing conditions by Ni-NTA–agarose affinity chromatography, according to protocol N°17 of the QIAexpressionist manual (5th edition, Qiagen). Protein fractions were analyzed by SDS–PAGE and Coomassie blue stained to determine the visibility of large protein complexes and dialyzed against storage buffer (150 mM NaCl, 50 mM Tris-HCl, pH 8.0, 0.1% Triton X-100). The crosslinking of the purified complexes was reversed by adding 2% SDS and heating at 95 °C for 20 min. Then, proteins were precipitated with trichloroacetic acid, pelleted by centrifugation, washed with cold acetone, and dried at room temperature for 20 min. Precipitated proteins (100 μg) were solubilized in 10 μl of water, reduced by with 2.5 μl of reduction buffer (45 mM DTT, 100 mM ammonium bicarbonate) for 30 min at 37 °C, and alkylated by adding 2.5 μl of alkylation buffer (100 mM iodoacetamide, 100 mM ammonium bicarbonate) for 20 min at 24 °C in dark. The peptide mixture was subjected to trypsin digestion and separated by LC-MS/MS, as previously described (45).

### Bacterial two-hybrid assay

Protein interactions between BarA and HflK or HflC were tested by a bacterial adenylate cyclase two-hybrid assay (42). Briefly, recombinant plasmids encoding proteins of interest fused to the T25 or T18 domain of adenylate cyclase were cotransformed into *E. coli* BTH101 (*cya*^−^) cells (Euromedex). Five single colonies from each transformant was grown in LB, supplemented with kanamycin and ampicillin, overnight at 30 °C. Then, cells were spot plated on LB agar supplemented with X-gal, 0.5 mM isopropyl-1-thio-β-D-galactopyranoside and antibiotics, or diluted into fresh LB medium with the same antibiotics and 0.5 mM isopropyl-1-thio-β-D-galactopyranoside, and grown at 30 °C to an *A*_600_ of 0.6 for the quantitative determination of β-galactosidase activity.

### Fluorescence microscopy

*E. coli* cells carrying either *hflK-mCherry*, *HflK-mCherry*, or *barA-mEyfp* were grown in LB medium at 37 °C and, at the indicated *A*_600_, 2 μl aliquots of the cell cultures were collected and immobilized on glass slides previously covered with freshly made M9 medium 1% agarose pads (70). The *barA-mEyfp* expression was slightly induced by adding 80 μM l-arabinose 20 min before samples were taken, and cells were immobilized. Cells were examined using a Leica DM6000B upright fluorescence microscope equipped with an oil-immersion objective lens microscope (100x/1.47). Yfp fluorescence was monitored using a K3 filter (excitation, BP 470–490 nm filter; DM510 nm; emission LP515 nm filter), and mCherry fluorescence was monitored using a N3 filter (excitation, BP 546–512 nm filter; DM564 nm; emission BL 600/40 nm filter). Fluorescence images were analyzed with ImageJ software (71) and subjected to background subtraction using a rolling ball radius of 20 pixels, and mCherry or Yfp fluorescence signals were colored in red or green, respectively.

### Electrophoretic gel mobility shift assays for CsrA-RNA binding

Binding of CsrA to RNAs was determined by EMSA with recombinant CsrA-His_6_, purified as described previously (20), and RNA synthesized *in vitro* with MEGAshortscript Kit (Ambion). The template DNA for *in vitro* transcription of sRNAs was generated by PCR from MG1655 genomic DNA, using primers *hflK* Fwd T7 and *hflK* Rev T7. *In vitro*-transcribed sRNAs were gel-purified, treated with Antarctic phosphatase (NEB), and radiolabeled at the 5′ end using [γ-^32^P] ATP and T4 polynucleotide kinase. Binding reactions contained 0.5 nM labeled RNA, 10 mM MgCl2, 100 mM KCl, 32.5 ng total yeast RNA, 20 mM DTT, 7.5% glycerol, 4 U SUPERasin (Ambion), and various concentrations of CsrA (0–400 nM) and incubated at 37 °C for 30 min. Reaction mixtures were separated on native polyacrylamide gels using 1X TBE as the electrophoresis buffer, gels dried and imaged using a PMI phosphorimager (Bio-Rad). The signals of free (F) and shifted/bound RNA-Protein (B) complex were quantified with the help of Quantity One software (Bio-Rad). A linear regression analysis of the data was performed to calculate the apparent equilibrium binding constant (Kd) for the RNA-protein interaction (Fig. S5). Data presented in the Fig. S5 are averages of two independent experiments.

### Determination of β-galactosidase activity

*E. coli* cells carrying the UvrY-P–activatable *csrB-lacZ* reporter were grown aerobically in LB adjusted to pH 7.0 and buffered with 100 mM Mops, at 37 °C. Cells carrying the ArcA-P–activatable *cydA-lacZ* operon fusion were grown aerobically in 10 ml of LB medium containing 100 mM Mops (pH 7.4) in 100-ml baffled flasks at 37 °C with shaking (300 rpm) or were grown anaerobically in a screw-capped tube filled with medium up to the rim at 37 °C and stirred by a magnet. Samples were withdrawn at mid-exponential growth (*A*_600_ of 0.6), and β-galactosidase activity was assayed and expressed in Miller units as described previously (72).

### Western blot analysis and protein stability determination

Cultures for Western blot analyses were grown aerobically at 37 °C and harvested by centrifugation at the indicated *A*_600_. Cultures for BarA half-life determination were grown aerobically in LB medium at 37 °C, at an *A*_600_ of 2,0, 20 μg/ml tetracycline were added, samples were withdrawn, and cells were harvested by centrifugation at the indicated times. For the BarA, HflK-HA, HflC-HA, and DnaK identification and semiquantification by immunoblotting, the cell pellets were resuspended in an appropriate volume of 2X Laemmli sample buffer and boiled for 5 min. Aliquots of 10 μl were separated by SDS-PAGE (10% polyacrylamide gel), and the proteins were transferred to a Hybond-ECL filter (Amersham Biosciences). The filter was equilibrated in TTBS buffer (25 mM Tris, 150 mM NaCl, and 0.05% Tween 20) for 10 min and incubated in blocking buffer (1% milk in TTBS) for 1 h at room temperature. BarA polyclonal antibodies, raised against His_6_-BarA^198–918^ (39), monoclonal antibodies against HA (Sigma Aldrich), or monoclonal antibodies against DnaK (Enzo Life Sciences) were added at a dilution of 1:10,000 and incubated for 1 h at room temperature. The bound antibody was detected by using anti-rabbit IgG antibody or anti-mouse IgG antibody conjugated to horseradish peroxidase (1:10,000 dilution) and the Immobilon Western detection system (Millipore). Protein bands were quantified using ImageJ software (71).

## Data availability

All data are contained in the article and in the Supporting information.

## Supporting information

This article contains supporting information (4, 6, 10, 39, 42, 44, 45, 46, 47, 61, 64, 65, 66, 67, 68).

## Conflict of interest

The authors declare that they have no conflicts of interest with the contents of this article.
