# Supplementary material for: Spatiotemporal regulation of the BarA/UvrY two-component signaling system
Source: J Biol Chem. 2023 May 17;299(6):104835. doi: 10.1016/j.jbc.2023.104835 (PMC10277598; doi:10.1016/j.jbc.2023.104835)
Supplement: Supporting Figures S1–S6 and Tables S1–S3 [file mmc1.docx]

Supplementary Information for

**Spatiotemporal regulation of the BarA/UvrY two-component signaling system**

Fernanda Urias Contreras, Martha I. Camacho, Archana Pannuri, Tony Romeo, Adrian F. Alvarez, and Dimitris Georgellis*

*Dimitris Georgellis

Email: dimitris@ifc.unam.mx

**This PDF file includes:**

Figure S1

Figure S2

Figure S3

Figure S4

Figure S5

Figure S6

Table S1

Table S2

Table S3

**Figure S1**


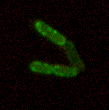

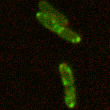

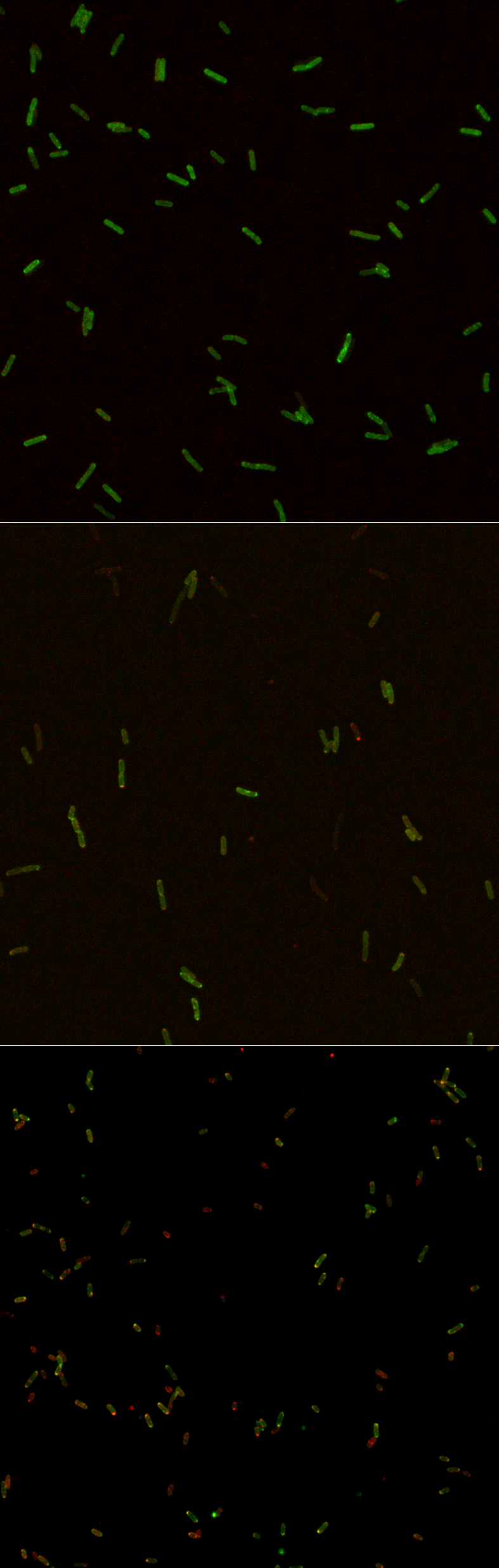


OD_600_ 0.4

OD_600_ 2.8

OD_600_ 1.2

Full FOV fluorescence image (BarA-Yfp / HflK-mCherry merge)

Magnified view of the selected regions

Cell growth

% of cells

(localization pattern)

75 % BarA non-polar localization

25 % BarA/HflK polar co-localization

90 % (BarA non-polar localization)

10% BarA polar localization

80 % BarA/HflK polar co-localization


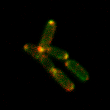


3 µm

3 µm

3 µm

5 µm

5 µm

5 µm

**Figure S1.** Full FOV fluorescence images and extended analysis of BarA and HflK localization /co-localization patterns.

**Figure S2**

**
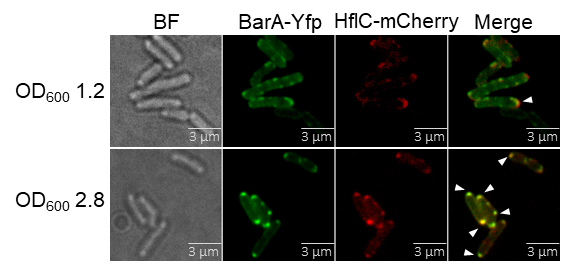
**

**Figure S2.** Co-localization analysis of BarA and HflC proteins. Representative fluorescence images of *E. coli* live cells expressing BarA-mEyfp (colored in green) and HflC-mCherry (colored in red) translational fusions, harvested during the early stationary phase (OD_600_ of 1.2) or during the stationary phase (OD_600_ of 2.8). Left panels show the bright field (BF) imaging of cells; right panels show the merged fluorescence signals, which appear yellow where the two fluorescence signals overlapped. Triangles indicate co-localized foci.

**Figure S3**

5’- A/C U/A/G G G A U/A/C G/U -3’ (47)

5’- R U A C A R G G A U G U -3’ (46)

5’- A A G **C** G **U** **G** **G** **A** G **U** C -3’ (*hflK* 1)

5’- C A **A** **C**  **A**  **G** **G** **G** **A**  **U** C A -3’ (*hflK* 2)

5’- A A **A** U **A** **U** **G** **G** **A**  G C A -3’ (*hflK* 3)

5’- U G G **C** G **U**  **G** **G** **A**  **A** **U** C -3’ (*hflK* 4)

5’- A A U A **A** C **G** **G** **A**  **C**  A A -3’ (*hflK* 5)

**Figure S3.** Comparison GGA and flanking sequences of *hflK* RNA with CsrA consensus sequences derived by CLIP-seq (47) and SELEX (46). The *hflK* 1-5 indicate the GGA sequences in the order they appear from the 5’ to 3’ direction (see m-fold structure prediction). Nucleotides in *hflK* RNA that match with either of the CsrA consensus are shown in bold letters.

**Figure S4**


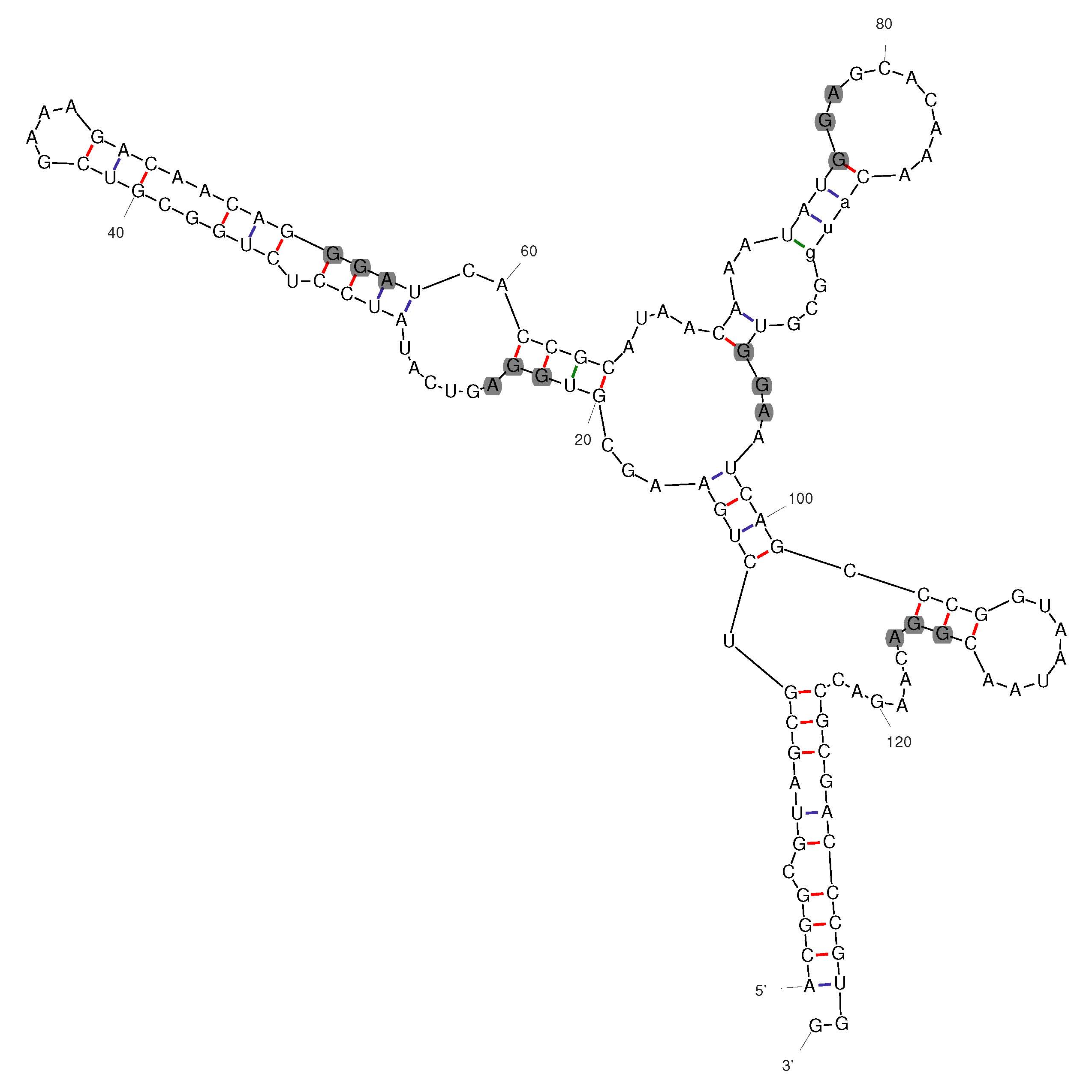


*hflK* 1

*hflK* 2

*hflK* 3

*hflK* 4

*hflK* 5

**Figure S4.** M-fold prediction of the 134 nt hflK RNA (ΔG = -33.69), extending from -86 to +48 nt with respect to the initiation codon. GGA sequences are shaded and the initiation codon is shown in lowercase letters.

**Figure S5**

Signal intensities of free and bound *hflK* RNA.

| **CsrA (nM)** | **Bound** | | **Unbound** | **Bound + unbound** | | **Bound/Total(f)** | | **1-f** | **f/(1-f)** | **logf/(1-f)** |
| --- | --- | --- | --- | --- | --- | --- | --- | --- | --- | --- |
| 0 | 0 | | 734427 | 734427 | | 0 | | 1 | 0 |  |
| 5 | 32198.3 | | 732341 | 764539 | | 0.042114698 | | 0.95789 | 0.04397 | -1.3569 |
| 10 | 50368.2 | | 722741 | 773109 | | 0.065150153 | | 0.93485 | 0.06969 | -1.1568 |
| 20 | 85867.5 | | 696152 | 782019 | | 0.109802308 | | 0.8902 | 0.12335 | -0.9089 |
| 35 | 284035 | | 487663 | 771698 | | 0.368064566 | | 0.63194 | 0.58244 | -0.2347 |
| 50 | 318161 | | 440706 | 758866 | | 0.419257735 | | 0.58074 | 0.72193 | -0.1415 |
| 70 | 533021 | | 237864 | 770885 | | 0.691440688 | | 0.30856 | 2.24087 | 0.35042 |
| 100 | 804384 | | 53006.8 | 857391 | | 0.938176665 | | 0.06182 | 15.1751 | 1.18113 |
| 200 | 828885 | | 11236.6 | 840122 | | 0.986625049 | | 0.01337 | 73.7666 | 1.86786 |
| 400 | 701412 | | 971.91 | 702383 | | 0.998616269 | | 0.00138 | 721.684 | 2.85835 |
|  |  | |  |  | |  | |  |  |  |
| f = Fraction of bound RNA | | | |  | |  | | |  |  |
| **CsrA (nM)** | | **logCsrA** | | | **logf/(1-f)** | |  |  |  |  |
| 5 | | 0.698970004 | | | -1.356879823 | |  |  |  |  |
| 10 | | 1 | | | -1.15682642 | |  |  |  |  |
| 20 | | 1.301029996 | | | -0.908874994 | |  |  |  |  |
| 35 | | 1.544068044 | | | -0.234748699 | |  |  |  |  |
| 50 | | 1.698970004 | | | -0.141502351 | |  |  |  |  |
| 70 | | 1.84509804 | | | 0.350416275 | |  |  |  |  |


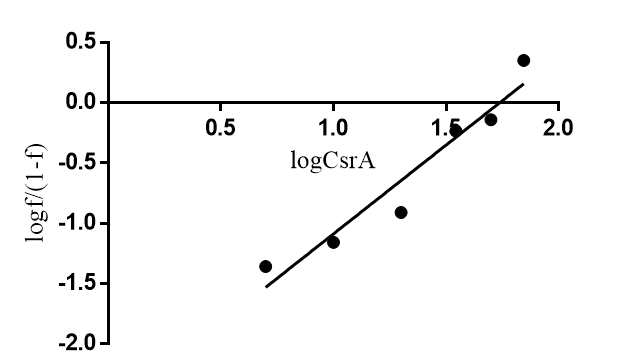


**Equation:**  Y = 1.473*X - 2.561

X intercept when Y is 0 = 1.738

Antilog of 1.738 = 54.7

**K_d_ = 54.7 or 55 nM**

**Figure S5.** Data and K_d_ calculations of *hflK* RNA binding to CsrA

**Figure S6**

1. **
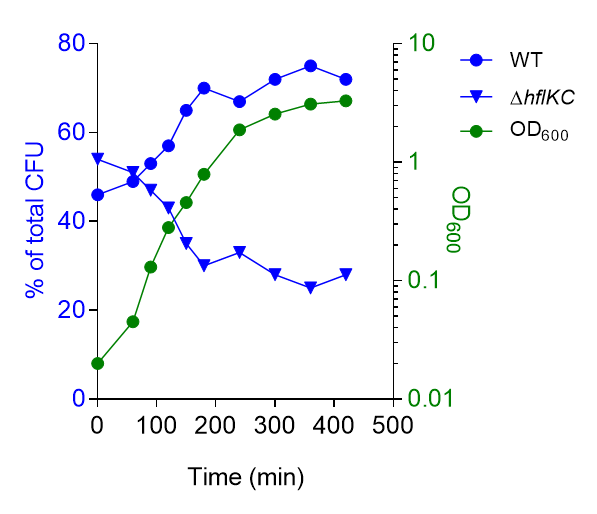

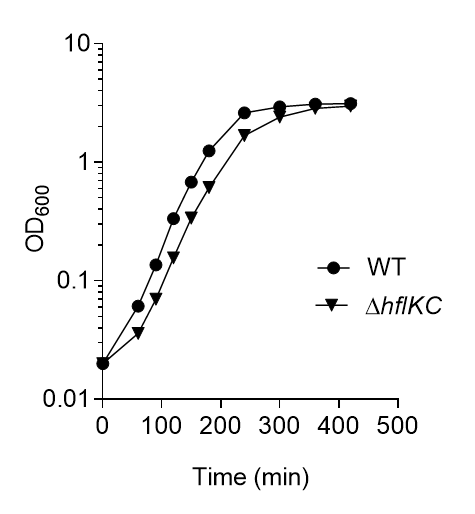
 B)**

**Figure S6.** Δ*hflKC* mutant exhibits impaired bacterial fitness. A) Cultures of KSB837 (WT) and IFC5047 (Δ*hflKC*::Kan^r^) strains were grown to the stationary phase and diluted with fresh LB medium to an OD_600_ of 0.01, and cell growth (OD_600_) were monitored for 420 min. B) Equal number of cells from stationary cultures of strains IFC5047 (Δ*hflKC*::Kan^r^) and KSB837 (wild type, Amp^r^) were mixed and used to inoculate fresh LB medium. Total cell growth (OD_600_), *hflKC* mutant strain growth (kanamycin resistant-CFUs/ml), and WT growth (ampicillin resistant-CFUs/ml) in the mixed culture were monitored for 420 min.

**Table S1.** Total spectra counts obtained by LC-MS/MS and protein assignment of His6-BarA pull-down and control samples

| **Entry name** | **Protein description** | **Molecular**  **Weight** | **Unique Peptide Count** | |
| --- | --- | --- | --- | --- |
|  |  |  | **Control** | **His6-BarA** |
| BARA_ECOLI | Signal transduction histidine-protein kinase BarA OS=Escherichia coli (strain K12) GN=barA PE=1 SV=1 | 102 kDa | 0 | 30 |
| ODO1_ECOLI | 2-oxoglutarate dehydrogenase E1 component OS=Escherichia coli (strain K12) GN=sucA PE=1 SV=1 | 105 kDa | 5 | 23 |
| SDHA_ECOLI | Succinate dehydrogenase flavoprotein subunit OS=Escherichia coli (strain K12) GN=sdhA PE=1 SV=1 | 64 kDa | 17 | 11 |
| ODP1_ECOLI | Pyruvate dehydrogenase E1 component OS=Escherichia coli (strain K12) GN=aceE PE=1 SV=2 | 100 kDa | 4 | 11 |
| EFTU2_ECOLI (+1) | Elongation factor Tu 2 OS=Escherichia coli (strain K12) GN=tufB PE=1 SV=1 | 43 kDa | 10 | 10 |
| ATPB_ECOLI | ATP synthase subunit beta OS=Escherichia coli (strain K12) GN=atpD PE=1 SV=2 | 50 kDa | 17 | 10 |
| ATPA_ECOLI | ATP synthase subunit alpha OS=Escherichia coli (strain K12) GN=atpA PE=1 SV=1 | 55 kDa | 15 | 7 |
| CRP_ECOLI | cAMP-activated global transcriptional regulator CRP OS=Escherichia coli (strain K12) GN=crp PE=1 SV=1 | 24 kDa | 2 | 7 |
| RL15_ECOLI | 50S ribosomal protein L15 OS=Escherichia coli (strain K12) GN=rplO PE=1 SV=1 | 15 kDa | 1 | 4 |
| ATPF_ECOLI | ATP synthase subunit b OS=Escherichia coli (strain K12) GN=atpF PE=1 SV=1 | 17 kDa | 10 | 3 |
| SLYD_ECOLI | FKBP-type peptidyl-prolyl cis-trans isomerase SlyD OS=Escherichia coli (strain K12) GN=slyD PE=1 SV=1 | 21 kDa | 2 | 3 |
| OMPA_ECOLI | Outer membrane protein A OS=Escherichia coli (strain K12) GN=ompA PE=1 SV=1 | 37 kDa | 9 | 3 |
| RNE_ECOLI | Ribonuclease E OS=Escherichia coli (strain K12) GN=rne PE=1 SV=6 | 118 kDa | 1 | 3 |
| NIKR_ECOLI | Nickel-responsive regulator OS=Escherichia coli (strain K12) GN=nikR PE=1 SV=1 | 15 kDa | 1 | 3 |
| RHLB_ECOLI | ATP-dependent RNA helicase RhlB OS=Escherichia coli (strain K12) GN=rhlB PE=1 SV=2 | 47 kDa | 0 | 3 |
| HFLK_ECOLI | Modulator of FtsH protease HflK OS=Escherichia coli (strain K12) GN=hflK PE=1 SV=1 | 46 kDa | 0 | 3 |
| GLMS_ECOLI | Glutamine--fructose-6-phosphate aminotransferase [isomerizing] OS=Escherichia coli (strain K12) GN=glmS PE=1 SV=4 | 67 kDa | 2 | 2 |
| LPP_ECOLI | Major outer membrane lipoprotein Lpp OS=Escherichia coli (strain K12) GN=lpp PE=1 SV=1 | 8 kDa | 2 | 2 |
| FRDA_ECOLI | Fumarate reductase flavoprotein subunit OS=Escherichia coli (strain K12) GN=frdA PE=1 SV=3 | 66 kDa | 6 | 2 |
| NUOCD_ECOLI | NADH-quinone oxidoreductase subunit C/D OS=Escherichia coli (strain K12) GN=nuoC PE=1 SV=3 | 68 kDa | 6 | 2 |
| SLYB_ECOLI | Outer membrane lipoprotein SlyB OS=Escherichia coli (strain K12) GN=slyB PE=2 SV=1 | 16 kDa | 2 | 2 |
| CYDA_ECOLI | Cytochrome bd-I ubiquinol oxidase subunit 1 OS=Escherichia coli (strain K12) GN=cydA PE=1 SV=1 | 58 kDa | 3 | 2 |
| RL19_ECOLI | 50S ribosomal protein L19 OS=Escherichia coli (strain K12) GN=rplS PE=1 SV=2 | 13 kDa | 1 | 2 |
| HFQ_ECOLI | RNA-binding protein Hfq OS=Escherichia coli (strain K12) GN=hfq PE=1 SV=2 | 11 kDa | 1 | 2 |
| MLAD_ECOLI | Probable phospholipid ABC transporter-binding protein MlaD OS=Escherichia coli (strain K12) GN=mlaD PE=1 SV=1 | 20 kDa | 1 | 2 |
| YFCH_ECOLI | Epimerase family protein YfcH OS=Escherichia coli (strain K12) GN=yfcH PE=3 SV=1 | 33 kDa | 0 | 2 |
| NUOI_ECOLI | NADH-quinone oxidoreductase subunit I OS=Escherichia coli (strain K12) GN=nuoI PE=1 SV=1 | 21 kDa | 1 | 2 |
| SELB_ECOLI | Selenocysteine-specific elongation factor OS=Escherichia coli (strain K12) GN=selB PE=1 SV=3 | 69 kDa | 0 | 2 |
| SDHB_ECOLI | Succinate dehydrogenase iron-sulfur subunit OS=Escherichia coli (strain K12) GN=sdhB PE=1 SV=1 | 27 kDa | 8 | 1 |
| SYE_ECOLI | Glutamate--tRNA ligase OS=Escherichia coli (strain K12) GN=gltX PE=1 SV=1 | 54 kDa | 1 | 1 |
| NARG_ECOLI | Respiratory nitrate reductase 1 alpha chain OS=Escherichia coli (strain K12) GN=narG PE=1 SV=4 | 140 kDa | 4 | 1 |
| RL5_ECOLI | 50S ribosomal protein L5 OS=Escherichia coli (strain K12) GN=rplE PE=1 SV=2 | 20 kDa | 1 | 1 |
| CYOA_ECOLI | Cytochrome bo(3) ubiquinol oxidase subunit 2 OS=Escherichia coli (strain K12) GN=cyoA PE=1 SV=1 | 35 kDa | 4 | 1 |
| RS3_ECOLI | 30S ribosomal protein S3 OS=Escherichia coli (strain K12) GN=rpsC PE=1 SV=2 | 26 kDa | 1 | 1 |
| NUOB_ECOLI | NADH-quinone oxidoreductase subunit B OS=Escherichia coli (strain K12) GN=nuoB PE=1 SV=1 | 25 kDa | 2 | 1 |
| HFLC_ECOLI | Modulator of FtsH protease HflC OS=Escherichia coli (strain K12) GN=hflC PE=1 SV=1 | 38 kDa | 3 | 1 |
| SERA_ECOLI | D-3-phosphoglycerate dehydrogenase OS=Escherichia coli (strain K12) GN=serA PE=1 SV=2 | 44 kDa | 1 | 1 |
| YHCB_ECOLI | Inner membrane protein YhcB OS=Escherichia coli (strain K12) GN=yhcB PE=1 SV=2 | 15 kDa | 4 | 1 |
| MBHM_ECOLI | Hydrogenase-2 large chain OS=Escherichia coli (strain K12) GN=hybC PE=1 SV=2 | 62 kDa | 5 | 1 |
| ACRA_ECOLI | Multidrug efflux pump subunit AcrA OS=Escherichia coli (strain K12) GN=acrA PE=1 SV=1 | 42 kDa | 3 | 1 |
| DLDH_ECOLI | Dihydrolipoyl dehydrogenase OS=Escherichia coli (strain K12) GN=lpdA PE=1 SV=2 | 51 kDa | 4 | 1 |
| FTSH_ECOLI | ATP-dependent zinc metalloprotease FtsH OS=Escherichia coli (strain K12) GN=ftsH PE=1 SV=1 | 71 kDa | 3 | 1 |
| RL2_ECOLI | 50S ribosomal protein L2 OS=Escherichia coli (strain K12) GN=rplB PE=1 SV=2 | 30 kDa | 0 | 1 |
| GYRB_ECOLI | DNA gyrase subunit B OS=Escherichia coli (strain K12) GN=gyrB PE=1 SV=2 | 90 kDa | 1 | 1 |
| RS5_ECOLI | 30S ribosomal protein S5 OS=Escherichia coli (strain K12) GN=rpsE PE=1 SV=2 | 18 kDa | 0 | 1 |
| YPDA_ECOLI | Sensor histidine kinase YpdA OS=Escherichia coli (strain K12) GN=ypdA PE=1 SV=1 | 63 kDa | 0 | 1 |
| WZZB_ECOLI | Chain length determinant protein OS=Escherichia coli (strain K12) GN=wzzB PE=1 SV=2 | 36 kDa | 1 | 1 |
| OXC_ECOLI | Oxalyl-CoA decarboxylase OS=Escherichia coli (strain K12) GN=oxc PE=1 SV=1 | 61 kDa | 0 | 1 |
| HEMX_ECOLI | Putative uroporphyrinogen-III C-methyltransferase OS=Escherichia coli (strain K12) GN=hemX PE=1 SV=1 | 43 kDa | 1 | 1 |
| DACA_ECOLI | D-alanyl-D-alanine carboxypeptidase DacA OS=Escherichia coli (strain K12) GN=dacA PE=1 SV=1 | 44 kDa | 2 | 1 |
| ATPD_ECOLI | ATP synthase subunit delta OS=Escherichia coli (strain K12) GN=atpH PE=1 SV=1 | 19 kDa | 2 | 1 |
| YFGM_ECOLI | UPF0070 protein YfgM OS=Escherichia coli (strain K12) GN=yfgM PE=3 SV=1 | 22 kDa | 3 | 1 |
| PTNAB_ECOLI | PTS system mannose-specific EIIAB component OS=Escherichia coli (strain K12) GN=manX PE=1 SV=2 | 35 kDa | 3 | 1 |
| SYT_ECOLI | Threonine--tRNA ligase OS=Escherichia coli (strain K12) GN=thrS PE=1 SV=1 | 74 kDa | 0 | 1 |
| KDUD_ECOLI | 2-dehydro-3-deoxy-D-gluconate 5-dehydrogenase OS=Escherichia coli (strain K12) GN=kduD PE=1 SV=2 | 27 kDa | 0 | 1 |
| RAPZ_ECOLI | RNase adapter protein RapZ OS=Escherichia coli (strain K12) GN=rapZ PE=1 SV=1 | 32 kDa | 0 | 1 |
| PLSB_ECOLI | Glycerol-3-phosphate acyltransferase OS=Escherichia coli (strain K12) GN=plsB PE=1 SV=2 | 91 kDa | 0 | 1 |
| YDEU_ECOLI | Uncharacterized protein YdeU OS=Escherichia coli (strain K12) GN=ydeU PE=5 SV=1 | 51 kDa | 0 | 1 |
| PPK_ECOLI | Polyphosphate kinase OS=Escherichia coli (strain K12) GN=ppk PE=1 SV=2 | 80 kDa | 0 | 1 |
| PPX_ECOLI | Exopolyphosphatase OS=Escherichia coli (strain K12) GN=ppx PE=1 SV=2 | 58 kDa | 0 | 1 |
| WBBI_ECOLI | Beta-1,6-galactofuranosyltransferase WbbI OS=Escherichia coli (strain K12) GN=wbbI PE=1 SV=1 | 38 kDa | 0 | 1 |
| CASC_ECOLI | CRISPR system Cascade subunit CasC OS=Escherichia coli (strain K12) GN=casC PE=1 SV=1 | 40 kDa | 0 | 1 |
| RECA_ECOLI | Protein RecA OS=Escherichia coli (strain K12) GN=recA PE=1 SV=2 | 38 kDa | 0 | 1 |
| **Without cytosolic proteins and/or those identified in the control assay** | | | | |
| BARA_ECOLI | Signal transduction histidine-protein kinase BarA OS=Escherichia coli (strain K12) GN=barA PE=1 SV=1 | 102 kDa | 0 | 30 |
| HFLK_ECOLI | Modulator of FtsH protease HflK OS=Escherichia coli (strain K12) GN=hflK PE=1 SV=1 | 46 kDa | 0 | 3 |
| YPDA_ECOLI | Sensor histidine kinase YpdA OS=Escherichia coli (strain K12) GN=ypdA PE=1 SV=1 | 63 kDa | 0 | 1 |
| PLSB_ECOLI | Glycerol-3-phosphate acyltransferase OS=Escherichia coli (strain K12) GN=plsB PE=1 SV=2 | 91 kDa | 0 | 1 |
| YDEU_ECOLI | Uncharacterized protein YdeU OS=Escherichia coli (strain K12) GN=ydeU PE=5 SV=1 | 51 kDa | 0 | 1 |
| PPK_ECOLI | Polyphosphate kinase OS=Escherichia coli (strain K12) GN=ppk PE=1 SV=2 | 80 kDa | 0 | 1 |
| PPX_ECOLI | Exopolyphosphatase OS=Escherichia coli (strain K12) GN=ppx PE=1 SV=2 | 58 kDa | 0 | 1 |

**Table S2.** Bacterial strains and plasmids used in this study

| Strain or plasmid | Relevant characteristics | Source or reference |
| --- | --- | --- |
| Strain |  |  |
| CF7789 | Wild type strain (WT); MG1655 Δ*lacZ* (MluI) | (10) |
| KSB837 | CF7789 λφ(*csrB’-lacZ*) | (10) |
| IFC5035 | KSB837 Δ*barA*::Kan^r^ | (6) |
| IFC5010 | KSB837 *csrA*::kan^r^ | (39) |
| BTH101 | Reporter strain for BACTH; *cya*-99 (adenylate cyclase mutant) | Euromedex |
| TR1-5CF7789 | CF7789 *csrA*::Kan^r^ | (4) |
| IFC5019 | CF7789 *hflC*:*ha*-Cm^r^ | (44) |
| IFC5021 | CF7789 *hflK*:*ha*-Cm^r^ | (45) |
| ECL5003 | MC4100 Δ*fnr*::Tn*9*(Cm^r^) λФ(*cydA*’*-lacZ*) | (64) |
| IFC5043 | CF7789 *hflK*:*mCherry*-Cam^r^ | This work |
| IFC5044 | CF7789 *hflC*:*mCherry*-Cam^r^ | This work |
| IFC5045 | CF7789 Δ*hflK*::Kan^r^ | This work |
| IFC5046 | CF7789 Δ*hflC*::Kan^r^ | This work |
| IFC5047 | CF7789 Δ*hflKC*::Kan^r^ | This work |
| IFC5048 | CF7789 Δ*hflK* | This work |
| IFC5049 | CF7789 Δ*hflC* | This work |
| IFC5050 | CF7789 Δ*hflKC* | This work |
| IFC5051 | CF7789 Δ*hflK* λφ(*csrB’-lacZ*) | This work |
| IFC5052 | CF7789 Δ*hflC* λφ(*csrB’-lacZ*) | This work |
| IFC5053 | CF7789 Δ*hflKC* λφ(*csrB’-lacZ*) | This work |
| IFC5054 | CF7789 *csrA*::Kan^r^ *hflK*:*ha*-Cm^r^ | This work |
| IFC5055 | CF7789 *csrA*::Kan^r^ *hflC*:*ha*-Cm^r^ | This work |
| IFC5056 | CF7789 Δ*hflKC* *csrA*::Kan^r^ λφ(*csrB’-lacZ*) | This work |
| IFC5057 | ECL5003 Δ*hflKC*::Kan^r^ | This work |
| IFC5058 | CF7789 Δ*hflKC* Δ*barA*::Kan^r^ λφ(*csrB’-lacZ*) | This work |
| Plasmid |  |  |
| pKD13 | Template plasmid for *kan* cassette amplification flanked by FRT sequences, Amp^R^, Kan^r^ | (61) |
| pKD4 | Template plasmid for *kan* cassette amplification flanked by FRT sequences, Amp^R^, Kan^r^ | (61) |
| pMX544 | *csrA* under native promoter in pEXT21, Sp^r^ | (39) |
| pMXFL2 | Template plasmid for generating C-terminal mCherry fusions to genes by λ-red recombination, Amp^r^, Cam^r^ | (44) |
| pACT3 | Low copy number vector, Cam^r^ | (66) |
| pMX020 | His_6_-ArcB^521-778^ under the control of *ara* promoter in pBAD30, Amp^r^ | (65) |
| pUC18-barA | *barA* in pUC18, Amp^r^ | (6) |
| pACT3-araP-arcB^521-778^ | His_6_-ArcB^521-778^ under the control of *ara* promoter in pACT3, Cam^r^ | This work |
| pMX559 | His_6_-BarA under control of *ara* promoter in pACT3, Cam^r^ | This work |
| pYFPC-4 | Integrative plasmid carrying mEyfp under the control of vanillate promoter, Gen^r^ | (67) |
| pUC19 | Cloning vector, Amp^r^ | (68) |
| pUC19-mEYFP | mEyfp in pUC19, Amp^r^ | This work |
| pUC19-barA-mEYFP | BarA-mEyfp translational fusion in pUC19, Amp^r^ | This work |
| pMX560 | BarA-mEyfp translational fusion under the control of *ara* promoter in pBAD30, Amp^r^ | This work |
| pKT25 | BACTH plasmid; pSU40-derivative encoding T25 fragment (residues 1–224) of CyaA under control of a *lac* promoter, Kan^r^ | (42) |
| pUT18C | BACTH plasmid; pUC19-derivative encoding T18 fragment (residues 225–399) of CyaA under control of *lac* promoter, Amp^r^ | (42) |
| pT25BarA | BarA N-terminal tagged with T25 fragment in pKT25, Kan^r^ | This work |
| pT25HflK | HflK N-terminal tagged with T25 fragment in pKT25, Kan^r^ | This work |
| pT18HflK | HflK N-terminal tagged with T18 fragment in pUT18C, Amp^r^ | This work |
| pT25ArcA | ArcA N-terminal tagged with T25 fragment in pKT25, Kan^r^ | This work |
| pT18ArcA | ArcA N-terminal tagged with T18 fragment in pUT18C, Amp^r^ | This work |
| pT25HflC | HflC N-terminal tagged with T25 fragment in pKT25, Kan^r^ | This work |
| pT18HflC | HflC N-terminal tagged with T18 fragment in pUT18C, Amp^r^ | This work |
| pMX561 | *hflKC* operon under *hfq-hflXKC* native promoter in pEXT21, Sp^r^ | This work |

**Table S3.** Oligonucleotides used in this study

| **Primer name** | **Primer sequence (5´-3')** | **Use** |
| --- | --- | --- |
| pFluor-hflK-Fw | CGCCAACGCGCAGCGTAACGACTACCAGCGTCAGGGGGAAATGGTGAGCAAGGGCGAG | Forward primer for *hflK* mCherry-labeling |
| pKD-hflK-Rv | CCAGCACGATGATGATAATCGCGATAACTGACTTACGCATATGAATATCCTCCTTAG | Reverse primer for *hflK* mCherry-labeling |
| pFluor-hflC-Fw | TTTCTTCCGCTACATGAAGACGCCGACTTCCGCAACGCGTATGGTGAGCAAGGGCGAG | Forward primer for *hflC* mCherry-labeling |
| pKD-hflC-Rv | AGGATGCGGTGGCTTTATTGACCTGTACCGCAGTCGTTATAATGAATATCCTCCTTAGTTC | Reverse primer for *hflC* mCherry-labeling |
| hflK70-Fw | CATAACAAATATGGAGCACAAACATGGCGTGGAATCAGCCCGGTAATAACGTGTAGGCTGGAGCTGCTTC | Forward primer for *hflK* and *hflKC* deletion |
| hflK70-Rv | GATAATCGCGATAACTGACTTACGCATCGTTATTCCCCCTGACGCTGGTAATTCCGGGGATCCGTCGACC | Reverse primer for *hflK* deletion |
| hflC70-Fw | TACCAGCGTCAGGGGGAATAACGATGCGTAAGTCAGTTATCGCGATTATCGTGTAGGCTGGAGCTGCTTC | Forward primer for *hflC* deletion |
| hflC70-Rv | ACATCCCTGAGGATGCGGTGGCTTTATTGACCTGTACCGCAGTCGTTATAATTCCGGGGATCCGTCGACC | Reverse primer for *hflC* deletion |
| hflC-pKD-Rv | GGATGCGGTGGCTTTATTGACCTGTACCGCAGTCGTTATATCCATATGAATATCCTCCTTAG | Reverse primer for *hflKC* deletion |
| Yfpcf1SacI | CAAGAGCTCATGGTGAGCAAGGGCGAGGAG | Forward primer for *mEyfp* amplification and cloning |
| YfPcr1HindIII | CAAAAGCTTCTTACTTGTACAGCTCGTCCATGC | Reverse primer for *mEyfp* amplification and cloning |
| barA-NdeI-Fw | CAACATATGACCAACTACAGCCTGCGC | Forward primer for *barA* amplification and cloning |
| barAr1SacI | CAAGAGCTCCCCGAGAATTTTGCTGGCTTCG | Reverse primer for *barA* amplification and cloning |
| hfqP-Fw-Hind | CCCAAGCTTCCCACTGTTAGTGGGCGG | Forward primer for amplification of *hfq-hflXKC-* operon promoter region |
| hfqP-Rv | CGGGATCCCATATGCTCTTTTCCTTATATGCTTATTTG | Reverse primer for amplification of *hfq-hflXKC*- operon promoter region |
| hflK-ORF-Fw | CCCCATATGGCATAACAAATATGGAGCACA | Forward primer for *hflKC* amplification and cloning |
| hflC-Rv | GGAATTCCCGACATCCCTGAGGATG | Reverse primer for *hflKC* amplification and cloning |
| DH-ACBarA-Fw | CGGGATCCCATGACCAACTACAGCCTG | Forward primer for *barA* amplification and construction of BACTH plasmids |
| ACBarA-Rv | CGGAATTCCGCCGCATCCGGCATAAAC | Reverse primer for *barA* amplification and construction of BACTH plasmids |
| DH-ACHflK-Fw | GGGATCCCATGGCGTGGAATCAGCC | Forward primer for *hflK* amplification and construction of BACTH plasmids |
| DH-ACHflK-Rv | CGGAATTCCCGCGCTCACCTTCTTTGAC | Reverse primer for *hflK* amplification and construction of BACTH plasmids |
| DH-ACHflC-Fw | CGGGATCCCATGCGTAAGTCAGTTATCGC | Forward primer for *hflC* amplification and construction of BACTH plasmids |
| DH-ACHflC-Rv | GCCAGATTGTCGAATTCATTC | Forward primer for *hflC* amplification and construction of BACTH plasmids |
| DH-ACArcA-Fw | CGGGATCCCATGCAGACCCCGCACATTCTTATC | Forward primer for *arcA* amplification and construction of BACTH plasmids |
| DH-ACArcA-Rv | CGGAATTCCGCGCTGTTGTTGGGAACC | Reverse primer for *arcA* amplification and construction of BACTH plasmids |
| *hflK* Fwd T7 | TAATACGACTCACTATAG ACGGCGTAGCGTCTGAAGC | Forward primer for generating the template for in vitro transcription of *hflK* RNA |
| *hflK* Rev T7 | CCACGGGTCGCGGTCTTGTC | Reverse primer for generating the template for in vitro transcription of *hflK* RNA |
